# *Arabidopsis thaliana* mTERF10 and mTERF11, but Not mTERF12, Are Involved in the Response to Salt Stress

**DOI:** 10.3389/fpls.2017.01213

**Published:** 2017-07-14

**Authors:** Duorong Xu, Dario Leister, Tatjana Kleine

**Affiliations:** Plant Molecular Biology, Department Biology I, Ludwig-Maximilians-Universität München Planegg-Martinsried, Germany

**Keywords:** Arabidopsis, chloroplast, nucleoid, mTERF, acclimation, stress, salt

## Abstract

Plastid gene expression (PGE) is crucial for plant development and acclimation to various environmental stress conditions. Members of the “mitochondrial transcription termination factor” (mTERF) family, which are present in both metazoans and plants, are involved in organellar gene expression. *Arabidopsis thaliana* contains 35 mTERF proteins, of which mTERF10, mTERF11, and mTERF12 were previously assigned to the “chloroplast-associated” group. Here, we show that all three are localized to chloroplast nucleoids, which are associated with PGE. Knock-down of *MTERF10, MTERF11*, or *MTERF12* has no overt phenotypic effect under normal growth conditions. However, *in silico* analysis of *MTERF10*, -*11*, and -*12* expression levels points to a possible involvement of mTERF10 and mTERF11 in responses to abiotic stress. Exposing mutant lines for 7 days to moderate heat (30°C) or light stress (400 μmol photons m^−2^ s^−1^) fails to induce a phenotype in *mterf* mutant lines. However, growth on MS medium supplemented with NaCl reveals that overexpression of *MTERF11* results in higher salt tolerance. Conversely, *mterf10* mutants are hypersensitive to salt stress, while plants that modestly overexpress *MTERF10* are markedly less susceptible. Furthermore, *MTERF10* overexpression leads to enhanced germination and growth on MS medium supplemented with ABA. These findings point to an involvement of mTERF10 in salt tolerance, possibly through an ABA-mediated mechanism. Thus, characterization of an increasing number of plant mTERF proteins reveals their roles in the response, tolerance and acclimation to different abiotic stresses.

## Introduction

Chloroplasts are of cyanobacterial origin (Raven and Allen, 2003) and harbor nowadays a reduced genome that mainly encodes proteins involved in photosynthesis and plastid gene expression (PGE). PGE is crucial for plant development and photosynthesis, but its regulation is only partially understood. This is largely because, although plastids still display characteristics of a prokaryotic-like structure of their genome, their gene expression machinery is much more elaborated compared to that of their cyanobacterial ancestor (reviewed in: Liere et al., 2011). Therefore, PGE requires plenty of proteins encoded in the nucleus that support transcription, splicing, trimming and editing of organellar RNAs, and regulate their translation (Schmitz-Linneweber and Small, 2008; Stern et al., 2010; Hammani et al., 2014; Tiller and Bock, 2014; Börner et al., 2015).

Also the nucleus-encoded proteins of the mitochondrial transcription termination factor (mTERF) family regulate mitochondrial and PGE at diverse levels (Kleine and Leister, 2015). The mTERF proteins have been identified in both plants and metazoans (Linder et al., 2005). Human mTERF1, which is the first characterized mTERF, is one of four mammalian mTERF proteins, and was identified nearly 30 years ago as a factor that acts on transcription termination in mitochondrial extracts (Kruse et al., 1989). Its presumptive function as a transcription terminator (of heavy-strand transcripts) gave the family its name. More recently however, models have been suggested in which mTERF1 acts chiefly as a terminator of antisense transcription (Terzioglu et al., 2013) or in polar replication fork arrest (Shi et al., 2016). The true molecular function of mouse mTERF2 also remains unclear, with some reports suggesting that it binds to the same mitochondrial DNA region as mTERF1 and mTERF3 (Wenz et al., 2009), while another contends that the DNA-binding activity of mTERF2 is not sequence-specific (Pellegrini et al., 2009). Knock-out of *Mterf3* in mice leads to embryonic lethality (Park et al., 2007), and conditional knockout of *Mterf3* in the heart has identified a novel role for its protein product in the biogenesis of metazoan mitochondrial ribosomes (Wredenberg et al., 2013). *Mterf4* knock-out mice are also embryonic lethal (Camara et al., 2011). Interestingly, human mTERF4 forms a complex with NSUN4, which is required for assembly of the small and large ribosomal subunits of the mitochondrial ribosome (Metodiev et al., 2014). Consequently, while the function for mTERF2 remains to be clarified, the remainder of the mammalian mTERFs do not support transcription termination, as it is suggested by their notation, but seem to take part in antisense transcription termination and ribosome biogenesis.

The number of mTERF family members has increased to approximately 30 throughout the evolution of land plants (Kleine, 2012), but information on their functions is only beginning to emerge. Most of the 35 *A. thaliana* mTERF proteins (mTERF1-mTERF35; Kleine, 2012) are localized to chloroplasts and/or mitochondria (Babiychuk et al., 2011), and seven of them (mTERF1, -4, -5, -6, -9, -15, and -18) have been functionally investigated in more detail (reviewed in: Kleine and Leister, 2015; Quesada, 2016). Essential functions of mTERF proteins in plant development are revealed by the effects of complete inactivation of three *MTERF* genes: *A. thaliana* mutants devoid of SOLDAT10 (SINGLET OXYGEN-LINKED DEATH ACTIVATOR10)/mTERF1 (Meskauskiene et al., 2009) or BSM (BELAYA SMERT)/RUG2 (RUGOSA2)/mTERF4 (Babiychuk et al., 2011; Quesada et al., 2011) are arrested in embryo development, and knock-out *mterf6-2* plants are albinotic and stop growing after 2 weeks (Romani et al., 2015). Moreover, the dissection of *mterf* mutants supports an involvement of plant mTERFs in responses to abiotic stress (reviewed in: Kleine and Leister, 2015; Quesada, 2016). Indeed, SOLDAT10 (Meskauskiene et al., 2009) and SUPPRESSOR OF *hot1–4* 1 (SHOT1; Kim et al., 2012) were isolated in forward genetic screens for loci that influence responses to abiotic stress. The *hot1-4* mutant is a dominant-negative allele of the heat-shock protein gene *HSP101*. SHOT1/mTERF18 is a mitochondrial protein and the *shot1-1* missense mutant and the *shot1-2* T-DNA insertion mutant each suppress the heat hypersensitivity of *hot1-4* plants. Moreover, other heat-sensitive mutant phenotypes are also suppressed by *shot1-2*, and *shot1-2* single mutants display a higher heat tolerance (Kim et al., 2012). SOLDAT10 is localized to chloroplasts, and plants homozygous for a weaker *soldat10* allele suffer from mild photo-oxidative stress already in low-light conditions; this results in turn in a stress acclimation response, which appears to confer improved hardiness against a combination of high-light and low-temperature stress (Meskauskiene et al., 2009). Other *mterf* mutants are also linked to stress responses. For example, *mda1* (*mterf5*), and *mterf9* seedlings are less susceptible to osmotic and salt treatments, which might be linked to their decreased sensibility to ABA (Robles et al., 2012, 2015). Furthermore, the *rug2-1* mutant is abnormally sensitive to temperature stress. At 26°C, *rug2-1* homozygotes undergo growth arrest, whereas at 16°C this growth phenotype is not expressed (Quesada et al., 2011).

A co-expression network for all *MTERF* genes (26 out of 35) which were present on the Affymetrix ATH1 genome array has been constructed (Kleine, 2012). The resulting clusters and information related to the subcellular locations of the proteins that are encoded by genes co-expressed with each *MTERF* gene were then used to assign the mTERFs into five groups, referred to as the “chloroplast,” “chloroplast-associated,” “mitochondrial,” “mitochondrion-associated,” and the “low expression” clusters.

In the present study, we characterized the members of the “chloroplast-associated” group, which comprises mTERF10 (AT2G34620), mTERF11 (AT3G18870), and mTERF12 (AT4G09620). The sub-chloroplast localization of mTERF10, -11, and -12 was defined by fluorescence microscopy of mTERF-GFP fusions and an RFP fusion protein (as a control for nucleoid localization). Lines with altered *MTERF10, MTERF11*, and *MTERF12* levels did not display phenotypes under normal growth conditions. *In silico* analyses with the eFP browser and Genevestigator were conducted, which pointed to an involvement of these three mTERFs in abiotic stress responses. To follow this up, the mutant lines were exposed to moderate heat (30°C), high light (400 μmol photons m^−2^ s^−1^), or salt (175 mM NaCl) stress, and subjected to ABA treatment. The *mterf10*, -*11*, and -*12* mutant lines responded to heat and high light stress like the wild type (WT). However, lack of mTERF10 or mTERF11 led to enhanced or reduced sensitivity to salt, respectively, while overexpression of *MTERF10* rendered seedlings more tolerant than WT to both salt and ABA.

## Materials and methods

### Plant material and growth conditions

The mutants *mterf10-1* (SAIL_12A03), *mterf10-2* (SALK_097699), *mterf11-1* (FLAG_357F09), *mterf11-2* (GABI_211D05), and *mterf12-1* (GABI_407E04) were identified in the SIGnAL database (Alonso et al., 2003), the *abi4-1* mutant was ordered from The European Arabidopsis Stock Centre (NASC; ID N8104). All mutants are in the Col-0 background except of *mterf11-1* which is a WS line.

*Arabidopsis thaliana* plants were grown in long-day conditions (16 h light/8 h dark) on potting soil (Stender). Plants were illuminated with HQI Powerstar 400 W/D lamps and a fluence rate of approximately 100 μmol photons m^−2^ s^−1^. To accomplish salt and ABA stress experiments, seedlings were grown on plant agar (Sigma-Aldrich) containing half-strength MS medium and 1.5% (w/v) sucrose at 22°C under 100 μmol photons m^−2^ s^−1^ provided by white fluorescent lamps under continuous light or long-day conditions. For salt stress experiments, MS medium was supplemented with 125 mM or 175 mM NaCl as indicated. For ABA experiments, MS medium was supplemented with 1 μM ABA.

### Nucleic acid extraction

For DNA isolation, leaf tissue was homogenized in extraction buffer containing 200 mM Tris/HCl (pH 7.5), 25 mM NaCl, 25 mM EDTA, and 0.5% (w/v) SDS. After centrifugation, DNA was precipitated by adding isopropyl alcohol. After washing with 70% (v/v) ethanol, the DNA was dissolved in distilled water.

For RNA isolation, frozen tissue was ground in liquid nitrogen. Following the addition of TRIZOL (Invitrogen) and chloroform according to the manufacturer's instructions, RNA was precipitated from the aqueous phase with isopropyl alcohol, then washed with 70% (v/v) ethanol, and dissolved in RNase-free water. Concentration and purity of RNA samples were determined spectroscopically in a GeneQuant pro RNA/DNA Calculator (GE Healthcare Europe GmbH). Isolated RNA was stored at −80°C until further use.

### cDNA synthesis and real-time PCR analysis

cDNA synthesis and real-time PCR analysis were performed as outlined before (Voigt et al., 2010). All reactions were done in triplicate on three biological replicates. The target genes and the respective primers, are listed in Supplementary Table S1. The *RCE1* gene was used as an internal reference in other studies (Voigt et al., 2010; Romani et al., 2015). *RCE1* transcript levels are not changed upon diverse conditions, especially not under diverse stress conditions including lincomycin and norflurazon treatment which affect organellar gene expression.

### RNAi, overexpression and intracellular protein localization

To reduce *MTERF12* mRNA levels by RNAi, a 145-bp fragment was amplified from genomic DNA with the primer pair AT4G09620-GST-attB1 and –attB2 (see Supplementary Table S1). The gel-purified PCR product was used for BP and LR Clonase reactions (GATEWAY Cloning; Invitrogen) which led to the final construct pB7GWIWG2/MTERF12 (for pB7GWIWG2, see Karimi et al., 2002). For overexpression and localization studies of mTERF10, mTERF11 and mTERF12, cDNAs encompassing the coding regions were amplified by PCR (see Supplementary Table S1 for primer information). Notably, in our Col-0 strain, *MTERF11* has an additional triplet (CAT; coding for histidine) inserted after nucleotide 27 (relative to the start codon) compared with the coding sequence from The Arabidopsis Information Resource (TAIR; www.arabidopsis.org). *MTERF10, MTERF11*, and *MTERF12* were cloned by GATEWAY technology (see above) into pB7FWG2 to generate fusions with enhanced GFP (eGFP), expression of which is controlled by the *Cauliflower mosaic virus* 35S promoter. For RAP-RFP fusions, the pENTR/RAP plasmid (Prof. Jörg Nickelsen, LMU Munich) was introduced into p2GWR7 by GATEWAY cloning. For overexpression of mTERF10, *MTERF10* was introduced by classical cloning with the NcoI restriction enzyme into pCAMBIA1302. For RNAi experiments with *MTERF12* and overexpression of mTERF10 and mTERF11, the plasmids pB7GWIWG2/MTERF12, pCAMBIA1302/MTERF10, and pB7FWG2/MTERF11 were independently transferred into *Agrobacterium tumefaciens*, and *A. thaliana* (ecotype Col-0 for *MTERF10* overexpression and *MTERF12* RNAi; ecotype WS for *MTERF11* overexpression) plants were transformed by the floral-dip method (Clough and Bent, 1998). After seed set, transgenic plants were selected on the basis of their resistance to BASTA (pB7GWIWG2/MTERF12 and pB7FWG2/MTERF11) or hygromycin (pCAMBIA1302/MTERF10), respectively.

For fluorescence visualization, leaves of 3-week-old Col-0 plants grown on MS medium were cut into small pieces and incubated for 16 h at 24°C in the dark in a protoplasting solution (10 mM MES, 20 mM CaCl_2_, 0.5 M mannitol (pH 5.8), 0.1 g ml^−1^ macerozyme (Duchefa), 0.1 g ml^−1^ cellulase (Duchefa). After isolation and transformation of protoplasts as described (Dovzhenko et al., 2003), preparations were examined with a Fluorescence Axio Imager microscope (Zeiss). Fluorescence was excited with the X-Cite Series 120 fluorescence lamp (EXFO) and images were collected at 500–550 nm (eGFP fluorescence), 570–640 nm (RFP fluorescence) and 670–750 nm (chlorophyll autofluorescence).

### Chlorophyll *a* fluorescence measurements

*In vivo* chlorophyll a fluorescence of whole plants was recorded using an imaging chlorophyll fluorometer (ImagingPAM, Walz GmbH, Effeltrich, Germany). Plants were dark adapted for 15 min and then exposed to a pulsed, blue measuring light (1 Hz, intensity 4) and a saturating light flash (intensity 5) to determine the maximum fluorescence F_m_ and the ratio (F_m_-F_0_)/F_m_ = F_v_/F_m_.

### Computational analyses

Protein sequences were retrieved from the National Center for Biotechnology Information (NCBI; http://www.ncbi.nlm.nih.gov/) and The Arabidopsis Information Resource (TAIR; http://www.arabidopsis.org). Amino acid sequences were aligned using the ClustalW program (http://www.ebi.ac.uk/clustalw; Chenna et al., 2003). The unrooted tree was constructed with the Phylip server Mobyle at the Pasteur Institute (http://mobyle.pasteur.fr/cgi-bin/portal.py#welcome).

## Results

### All members of the chloroplast-associated mTERF cluster are localized to nucleoids

**Figure 1:**
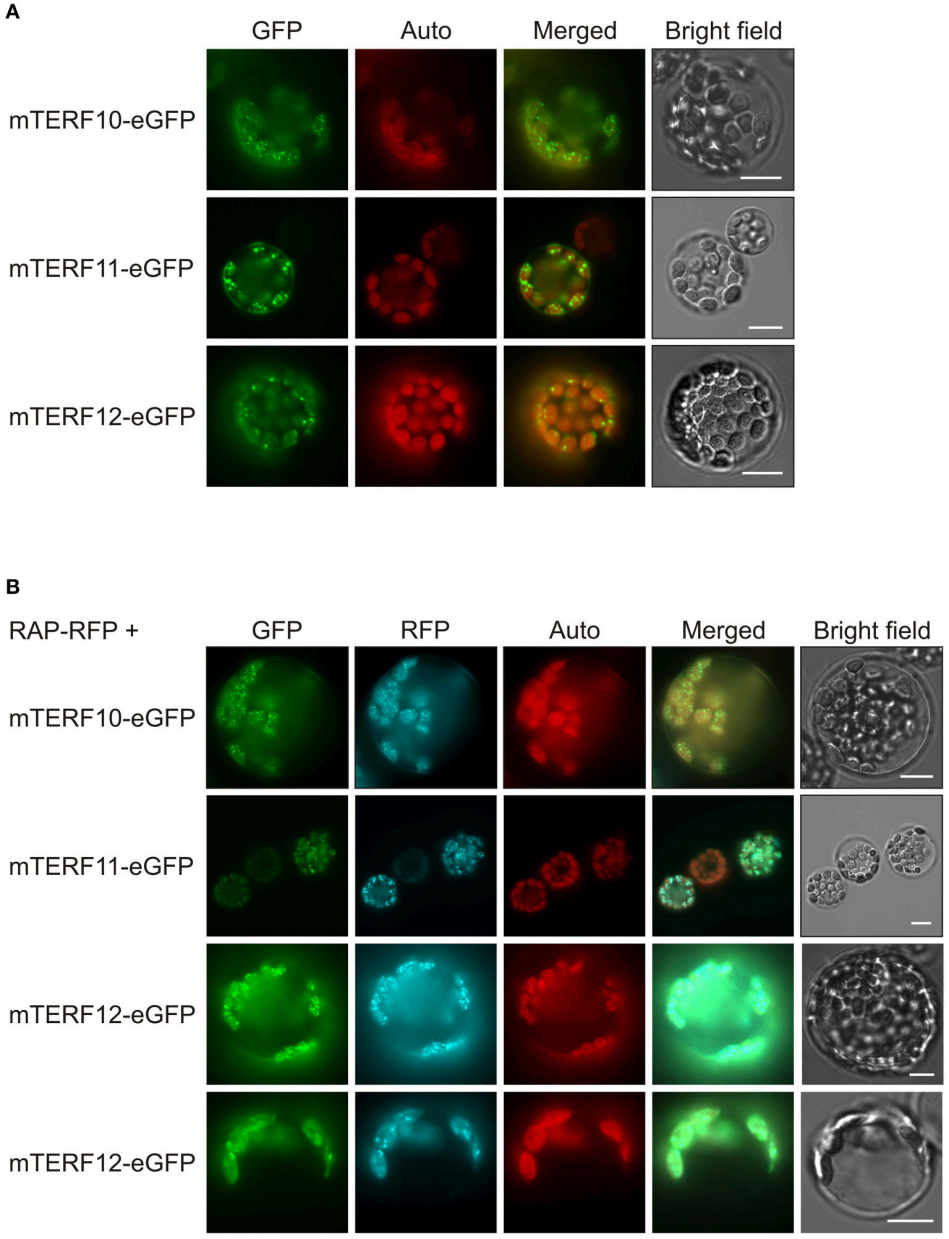
Localization of mTERF10, mTERF11 and mTERF12. **(A)** Fluorescence microscopy of *A. thaliana* protoplasts transiently expressing mTERF10, mTERF11 or mTERF12 fused to eGFP (mTERF10-eGFP, mTERF11-eGFP and mTERF12-eGFP). **(B)** To visualize nucleoids, protoplasts were co-transformed with a RAP-RFP fusion in combination with mTERF10-eGFP, mTERF11-eGFP, or mTERF12-eGFP. The eGFP fluorescence is shown in green (GFP), RFP fluorescence in cyan (RFP), autofluorescence of chloroplasts in red (Auto). The scale bars correspond to 10 μm.

The localizations of almost all *A. thaliana* mTERF proteins have been investigated by fluorescence microscopy of mTERF-GFP fusions transiently expressed in isolated protoplasts, and in guard cells of transgenic plants (Babiychuk et al., 2011). These data indicated that mTERF10, -11, and -12 are targeted to chloroplasts. To confirm these results and if possible define the precise locations of the proteins within the chloroplast, the eGFP fluorescence of mTERF10-, mTERF11-, or mTERF12-eGFP fusions, transiently overexpressed in Col-0 protoplasts, was monitored. Localization of all three fusion proteins to chloroplasts was confirmed (Figure 1A). However, the fluorescence signals were not uniformly distributed, but appeared as small dots in the chloroplasts. The size and distribution of these dots were suggestive of nucleoids, which are associated with PGE (Majeran et al., 2012). Localization of the *A. thaliana* RNA-binding protein RAP to nucleoids was previously established using a transiently expressed RAP-eGFP fusion (Kleinknecht et al., 2014). Therefore, Col-0 protoplasts were co-transformed with a RAP-RFP fusion in combination with mTERF10-eGFP, mTERF11-eGFP, or mTERF12-eGFP. Indeed, for each mTERF-eGFP construct, signals were found in dots together with the RFP signal. Merging of both signals confirmed colocalization of the mTERF10, -11, and -12 fusions with RAP, and therefore localization of all three mTERFs to nucleoids (Figure 1B). It is noteworthy, that especially mTERF12—and a minor fraction of RAP—tend to be localized in the chloroplast stroma when both mTERF12-eGFP and RAP-RFP are expressed together in protoplasts.

### Identification and phenotypic analysis of mutants for the *MTERF10, MTERF11, MTERF12* loci

**Figure 2:**
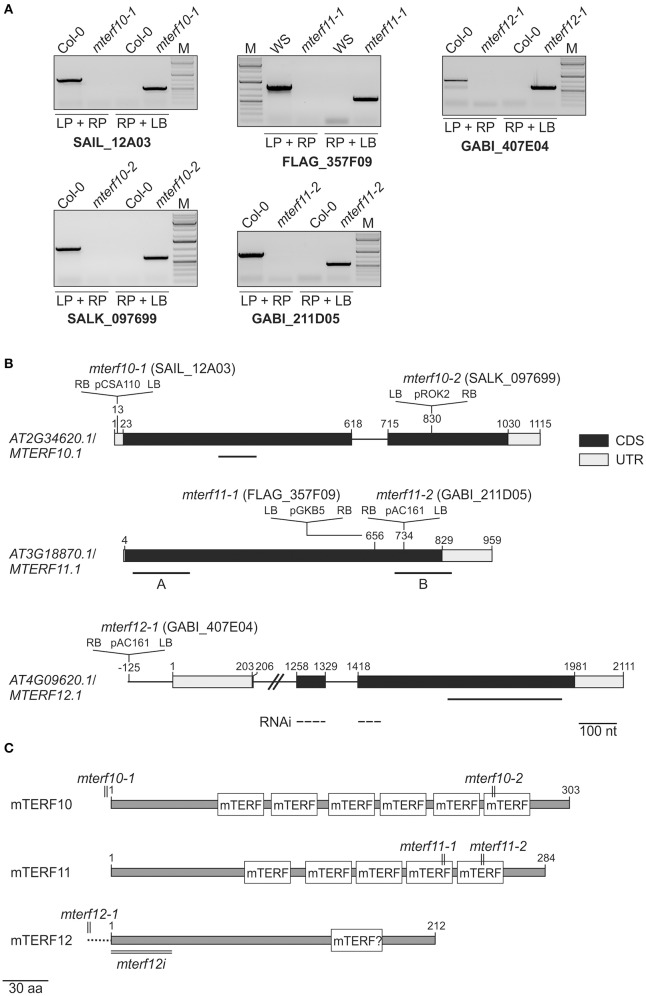
Identification of *mterf10, mterf11* and *mterf12* T-DNA insertion mutants, and generation of *MTERF12* RNAi lines. **(A)** Confirmation and identification of homozygous T-DNA insertions in the different *mterf* mutant lines. The combination of the gene-specific left and right primers (LP and RP) was used for amplification of sequences around the T-DNA insertion. The combination of RP and T-DNA left border primer (LB) was used for the verification of the T-DNA insertion. **(B)** Schematic representation and T-DNA tagging of the *MTERF10* (*AT2G34620*), *MTERF11* (*AT3G18870*), and *MTERF12* (*AT4G09620*) loci. Exons (black boxes), introns (black lines) and the 5′ and 3′ UTRs (gray boxes) are shown. Numbers are given relative to the transcription start site of the gene loci. Locations and orientation of T-DNA insertions are indicated, as deduced from RP + LB PCR products shown in **(A)** which were subsequently sequenced. Note that the insertions are not drawn to scale. Furthermore, the location of the *MTERF12* RNAi-directed sequence is indicated as a dashed line. **(C)** Schematic representation of mTERF10, mMTERF11, and mTERF12 proteins. The numbers and locations of mTERF domains are shown as white boxes. The relative positions of T-DNA and RNAi tagging are indicated.

To obtain insight into the physiological functions of mTERFs 10, 11, and 12, T-DNA insertion lines were identified in the SIGnAL database (Alonso et al., 2003). The insertions were confirmed by PCR (Figure 2A) and homozygous lines were selected. In the mutants *mterf10-1* (SAIL_12A03) and *mterf10-2* (SALK_097699) the T-DNAs are inserted in the 5′ UTR and the second exon, respectively (Figure 2B). The *mterf11-1* (FLAG_357F09) and *mterf11-2* (GABI_211D05) mutants both have their T-DNA insertion in the gene's single exon. For *MTERF12*, only one insertion line could be identified (*mterf12-1*, GABI_407E04), which contains a T-DNA in the promoter region (Figure 2B). To repress the *MTERF12* gene by RNAi, Col-0 lines were generated that contained constructs with an inverted repeat of a fragment spanning the first exon and a part of exon 2 of *MTERF12* (Figure 2A) which was under control of the constitutive *Cauliflower mosaic virus* 35S promoter.

Figure 2C shows the numbers and distributions of mTERF domains in the mTERF10, -11, and -12 proteins. In mTERF10 and mTERF11, six and five mTERF motifs are predicted by the Simple Modular Architecture Research Tool SMART (http://smart.embl-heidelberg.de/). One mTERF motif has been predicted for mTERF12 (our previous results, and see also Supporting Information of Babiychuk et al., 2011), but this domain is not annotated anymore with confidence by the SMART tool (http://smart.embl-heidelberg.de/smart/show_motifs.pl?ID=Q93ZZ2_ARATH). Thus, the classification of mTERF12 as an mTERF protein must be regarded as uncertain.

**Figure 3:**
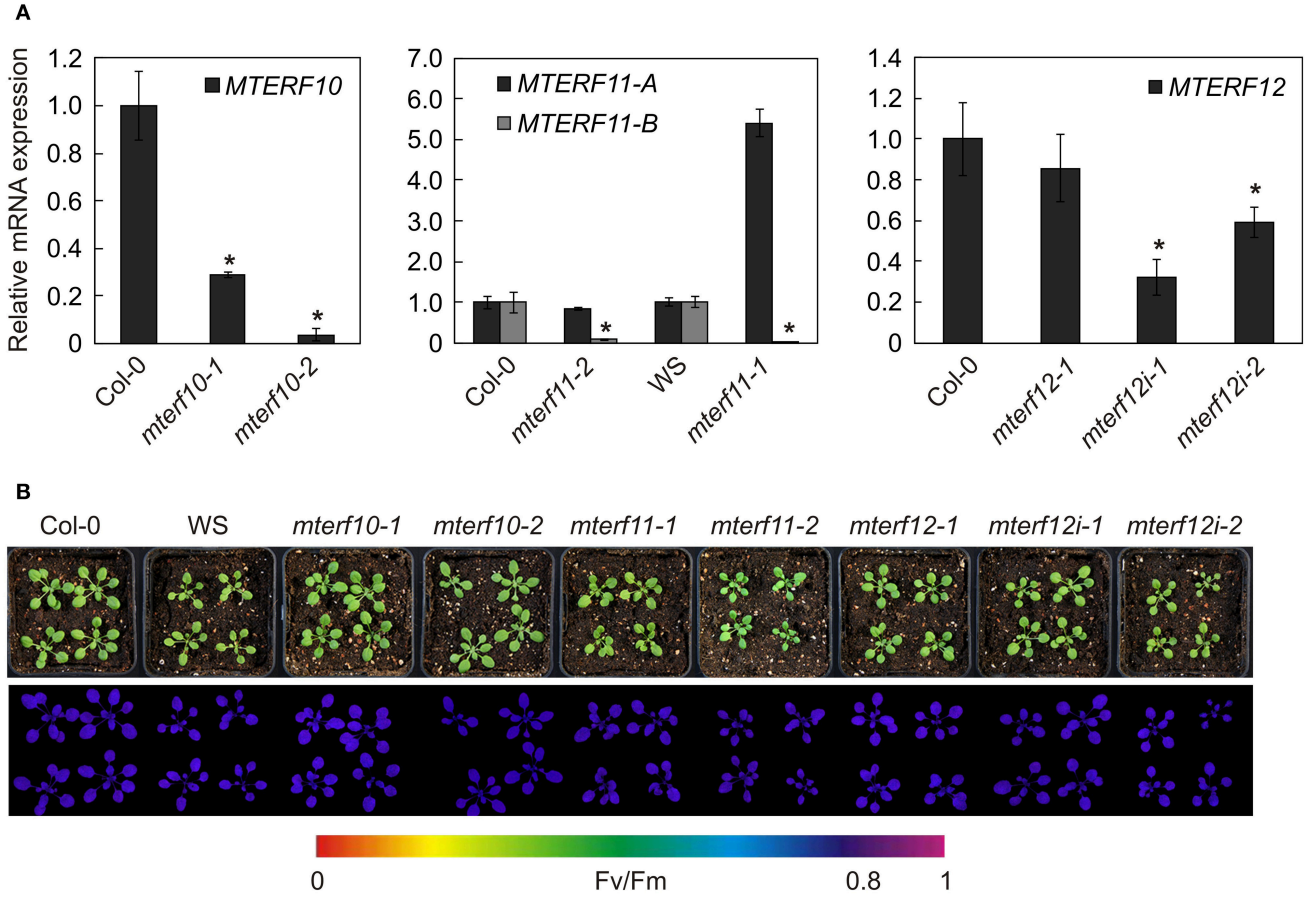
Characterization of *mterf10, mterf11, mterf12* T-DNA insertion and *MTERF12* RNAi (*mterf12i*) lines. **(A)** Real-time PCR analysis of *MTERF10, MTERF11, and MTERF12* mRNA levels. Real-time PCR was performed with primers specific for fragments indicated by horizontal black lines below the corresponding gene in Figure 2B. Expression values are reported relative to the corresponding transcript levels in Col-0. The results were normalized with respect to the expression level of *At4g36800*, which codes for a RUB1-conjugating enzyme (*RCE1*). Bars indicate standard deviations. Statistically significant differences (*t*-test; *p* < 0.05) between wild-type and mutant samples are indicated by an asterisk. **(B)** Phenotypes of 3-week-old wild-type (WS for *mterf11-1* and Col-0 for the remaining mutant lines) and mutant plants grown under long-day (16/8 h) light conditions. The maximum quantum yield of PSII (F_v_/F_m_) was measured with an ImagingPAM fluorometer.

All mutants are in the Col-0 background except of *mterf11-1* which is a WS line. Hence, in all following experiments, *mterf11-1* was compared with WS, while Col-0 was used as the WT standard for the other lines. Real-time PCR analysis was employed to determine the extent of repression of *MTERF* transcripts in the different mutant lines (Figure 3A). In 3-week-old *mterf10-1* and *mterf10-2* plants, *MTERF10* transcript levels were reduced to 29 and 4% of WT, respectively. To determine *MTERF11* transcript levels, primer pair A was chosen to detect transcripts initiated 5′ of the T-DNA insertions (Figure 2A). Using this set-up, *MTERF11* transcript levels were found to be unchanged (*mterf11-1*) and nearly 6-fold induced (*mterf11-2*) relative to their WT (Figure 3A). In the *mterf11-1* allele (which is FLAG_357F09), the T-DNA of the pGKB5 vector integrated in the 5′LB–T-DNA–RB3′ direction. It is of note here that the pGKB5 vector used to generate the FLAGdb T-DNA insertion line collection contains the 35S promoter on the LB side (Samson et al., 2002). The 35S promoter drives the expression of *PHOSPHINOTRICIN ACETYL TRANSFERASE* (*PAT*) used to select transgenic plants, and the *PAT* transcripts are terminated by the G7 terminator. It was already shown with two independent FLAG lines as an example that the G7 terminator can be an inefficient terminator in the context of the pGKB5 vector, allowing transcription to continue through and beyond the terminator sequence (Ulker et al., 2008). However, real-time PCR carried out with a primer pair covering the region 3′ of the T-DNA insertion detected greatly reduced *MTERF11* transcript levels in the *mterf11* mutants: 0.09% of WT in *mterf11-1* and 0.01% in *mterf11-2* (Figure 3A). *MTERF12* transcript levels were not affected in the *mterf12-1* mutant (Figure 3A). Therefore, *MTERF12* RNAi lines were tested for their ability to repress *MTERF12* gene expression. Six independent lines were screened, but the most effectively repressed lines, *mterf12i-1* and *mterf12i-2*, still retained 32% and 59% of WT (Col-0) amounts of *MTERF12* transcripts, respectively (Figure 3A). Under normal growth conditions, all identified mutant lines were phenotypically indistinguishable from WT (Figure 3B). To look for subtle photosynthetic phenotypes, the maximum quantum yield of photosystem II (F_v_/F_m_) was measured in Col-0, WS and all *mterf* mutants (Figure 3B), but no deviations in this parameter were detected in the mutants.

To summarize, the expression of all mTERF motifs should be strongly reduced in the *mterf10* mutants (particularly *mterf10-2*), while the *mterf11* mutants produce truncated transcripts. Assuming the latter are translated, the protein products would lack the last two mTERF domains (*mterf11-1*) or mTERF domain 5 only (*mterf11-2*) (Figure 2C). In the *mterf12i* lines, transcripts including the single putative mTERF domain were—at best—reduced to one-third of Col-0 levels. At all events, none of the *mterf* mutant lines display any obvious phenotype under normal growth conditions.

### Phylogenetic position of the mTERF10, -11, and -12 proteins

**Figure 4:**
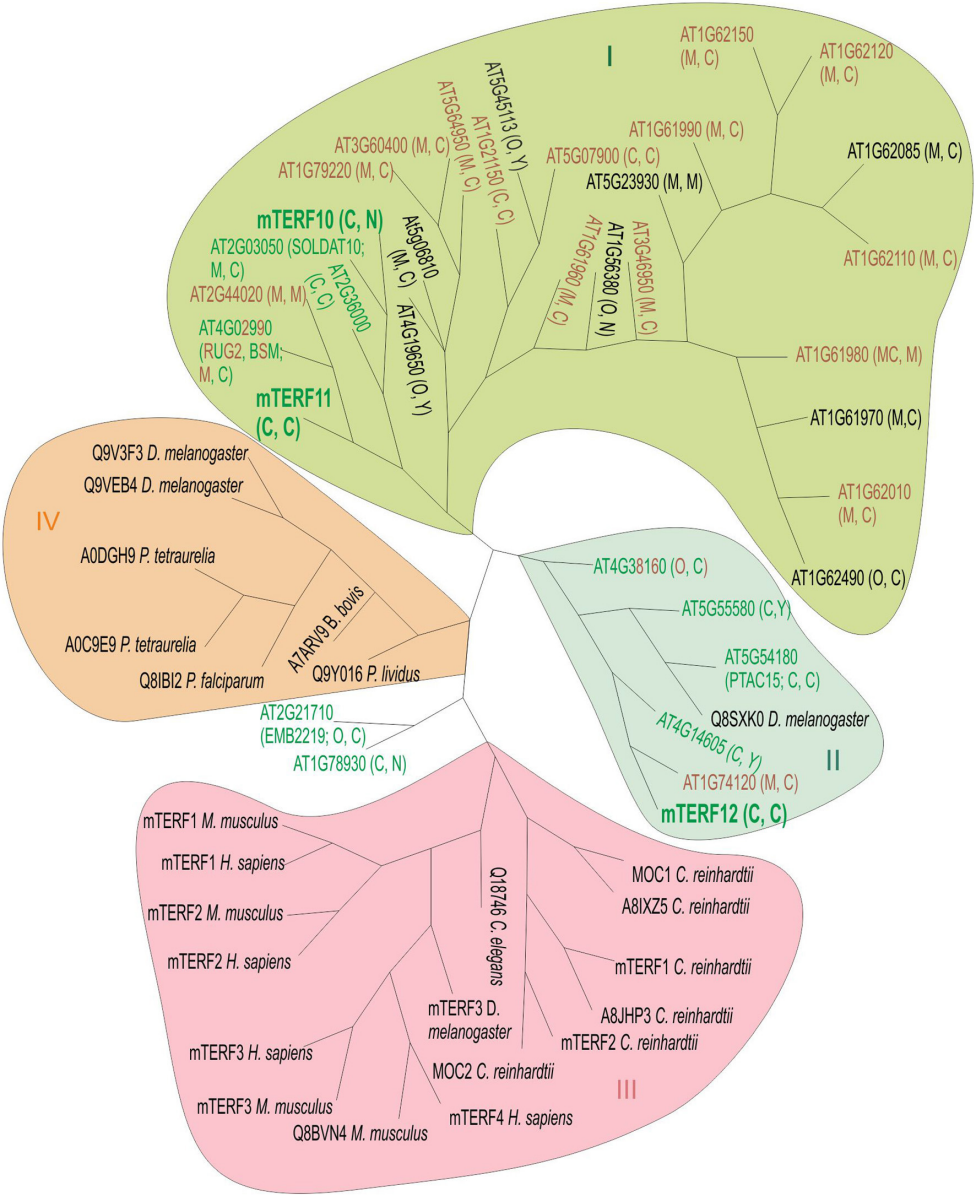
Unrooted phylogenetic tree of mTERF proteins. The tree is based on the amino acid sequences of 35 *A. thaliana* mTERF proteins and 23 other mTERF proteins from *Chlamydomonas reinhardtii* (*C. reinhardtii* MOC1 [Q8LJS6], MOC2 [A8IC10], mTERF1 [E1VD13], mTERF2 [E1VD14], A8IXZ5, A8JHP3), *Drosophila melanogaster* (*D. melanogaster* Q8SXK0, Q9V3F3, Q9VEB4, mTERF3 [Q06YR8]), *Homo sapiens* (*H. sapiens* mTERF1 [Q99551], mTERF2 [Q49AM1], mTERF3[Q96E29], and mTERF [Q7Z6M4]), *Mus musculus* (*M. musculus* mTERF1 [Q8CHZ9], mTERF2 [Q8BKY8], mTERF3 [Q8R3J4], Q8BVN4), *Caenorhabditis elegans* (*C. elegans* Q18746C), *Plasmodium falciparum* (*P. falciparum* Q8IBI2), *Babesia bovis* (*B. bovis* A7ARV9), *Paracentrotus lividus* (*P. lividus* Q9Y016) and *Paramecium tetraurelia* (*P. tetraurelia* A0DGH9). Green, brown and green-brown lettering depicts targeting to chloroplasts, mitochondria or dual targeting to chloroplasts and mitochondria, respectively, as reported elsewhere (Meskauskiene et al., 2009; Babiychuk et al., 2011; Quesada et al., 2011; Romani et al., 2015) and in this article. Letters in parentheses indicate predicted localization by TargetP (http://www.cbs.dtu.dk/services/TargetP) and WoLF PSORT (http://wolfpsort.org). Sequences were aligned by the ClustalW program (see Materials and Methods). The Phylip server Mobyle (see Methods) was used for phylogenetic tree constructions and comparison of distances (model: Jones-Taylor-Thornton matrix), employing a boostrap test with 1,000 replicates. Phylogenetic inference supports the existence of four main clades (I–IV). Clade I encompasses proteins encoded by a tandem gene cluster on *A. thaliana* chromosome 1 and several other *A. thaliana* mTERF proteins. In clade III, *C. reinhardtii* mTERFs are grouped together with animal mTERF proteins. Clade IV comprises mTERF proteins from diverse species including paramecium, sea urchin (*P. livides*), parasites (*P. falciparum* and *B. bovis*) together with mTERFs from Drosophila. The mTERF proteins mTERF10 and -11 (highlighted in large, bold letters) form clade I together with 25 other mTERF proteins, while mTERF12 (also highlighted in big, bold letters) is assigned to clade II, together with five other *A. thaliana* mTERFs and one Drosophila mTERF. C, chloroplast; M, mitochondrion; N, nucleus; Y, cytosol; O, other.

Because the *mterf10, -11*, and -*12* mutant lines lacked a clear phenotype under normal growth conditions (Figure 3), we asked whether this might be attributable to functional redundancy within the mTERF family. Several *MTERF* genes have undergone tandem duplications (on chromosome 1) and one block duplication (*AT4G19650* and *AT5G45113*; Kleine, 2012). But neither *MTERF12* nor *MTERF10* or *MTERF11* originated from a duplication event, so we can exclude the possibility of protein redundancy arising from gene duplication. To obtain an impression of the overall degree of sequence similarity within the mTERF protein family, we constructed a phylogenetic tree which included in addition to *A. thaliana* mTERFs, mTERF members from the green alga *Chlamydomonas reinhardtii, Homo sapiens, Mus musculus, Drosophila melanogaster*, and other organisms (Figure 4). The tree reveals four main clades. The mTERF members of *C. reinhardtii, H. sapiens* and *M. musculus* are all in the same clade. The majority of *A. thaliana* mTERFs form clade I which includes mTERF10 and -11, while mTERF12 along with five other *A. thaliana* mTERFs and one *D. melanogaster* mTERF constitute clade II. The mTERF10, -11, and -12 proteins are most closely related to mTERF1/SOLDAT10 (Meskauskiene et al., 2009), mTERF4/BSM/RUG (Babiychuk et al., 2011; Quesada et al., 2011) and mTERF15 (Hsu et al., 2014) proteins. Mutants for each of these three display phenotypes under normal growth conditions and have been shown to be involved in PGE or mitochondrial gene expression. Moreover, levels of sequence identity/similarity between mTERF10 and mTERF1 (38/68% over a stretch of 240 amino acids), mTERF11 and mTERF4 (26/41% over a stretch of 222 amino acids) and mTERF12 and mTERF15 (29/53% over a stretch of 77 amino acids), respectively, are noteworthy for the mTERF10/mTERF1 pair, but negligible for the other two.

### Changes in *MTERF* transcript levels in response to abiotic stresses

**Figure 5:**
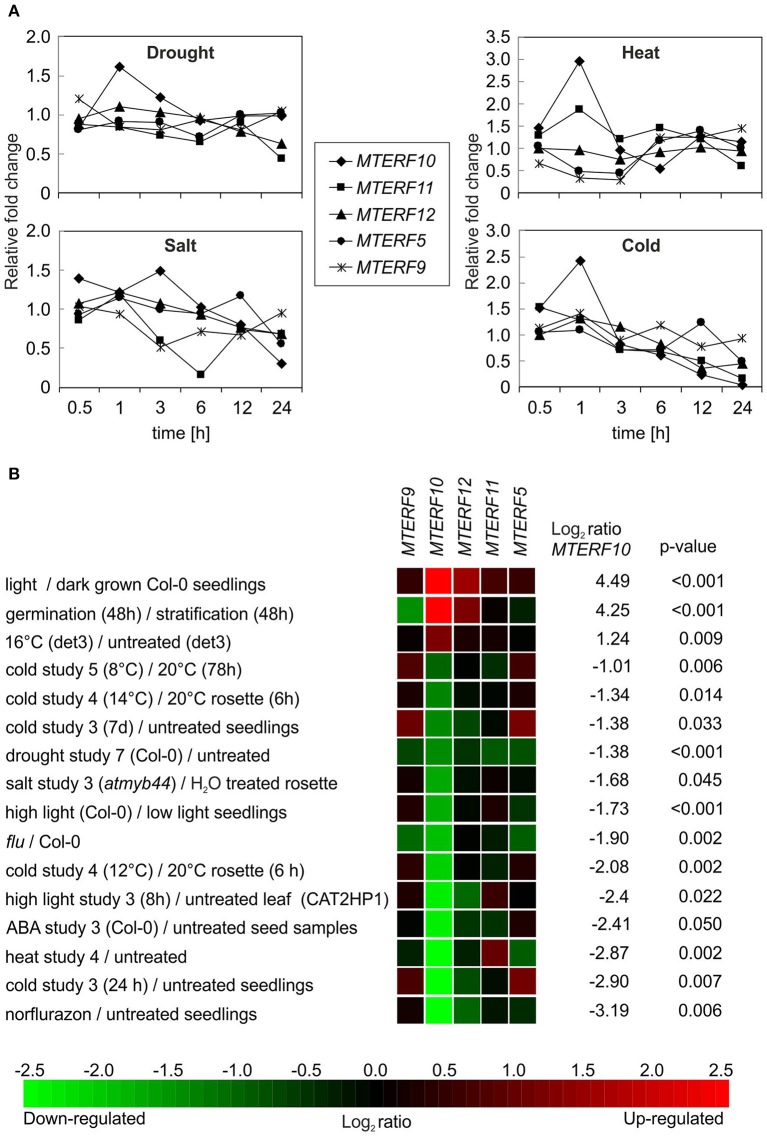
*In silico* analyses of changes in levels of *MTERF* transcripts in response to abiotic stresses. **(A)** *MTERF* transcript levels were extracted from the Arabidopsis eFP Browser (http://bar.utoronto.ca/efp/cgi-bin/efpWeb.cgi) with “Abiotic Stress” as a data source (Winter et al., 2007). Plant material from stress-treated and control plants was analyzed over a time course of 24 h. Here, the expression values are reported relative to the corresponding transcript levels in control conditions. **(B)** The Genevestigator Perturbations Tool (https://genevestigator.com/gv; Hruz et al., 2008) was applied to all available *A.thaliana* microarrays in combination with the 2-fold change filter and a *p*-value of < 0.05. Shown here is a selection of conditions related to abiotic stresses. Conditions were ordered according to the magnitude of the relative change in *MTERF10* mRNA (from high to low). An overview of all transcriptional responses of *MTERF10, MTERF11*, and *MTERF12* to perturbations can be found in Supplementary Figures S1–S3.

To gain deeper insights into the functions of mTERF10, -11, and -12, their mRNA expression patterns were analyzed. Co-expression analysis of 26 *MTERF* genes and their corresponding gene ontology annotations have already been reported (Kleine, 2012). However, that study was designed to provide a global classification. Hence subsequent Genevestigator analyses only dealt with the numbers of conditions/treatments that altered *MTERF* gene expression. In the present study, we extracted *MTERF* transcript levels from the Arabidopsis eFP browser (http://bar.utoronto.ca/efp/cgi-bin/efpWeb.cgi) with “Abiotic Stress” as a data source (Winter et al., 2007). In these experiments, 18-day-old plants were subjected to different stresses, and samples were taken over a time course of 24 h from stress-treated and control plants. We calculated the relative changes in *MTERF10*, -*11*, and -*12* transcript levels from plants exposed to drought, high salt, heat, or cold compared to control conditions (Figure 5A). Because *mda1* (*mterf5*) and *mterf9* mutants are known to exhibit altered stress responses (Robles et al., 2012, 2015), *MTERF5* and -*9* were included for reference. Under drought and heat stress, transcript levels of all investigated *MTERF* genes were only moderately changed (Figure 5A). With a 3-fold rise after 1 h of heat stress (MTERF10) and an approximately 0.3-fold change (*MTERF5* and *9*), those transcripts were the most responsive. Under salt and cold treatment, *MTERF* transcript levels tended to be reduced. Under both salt and cold stress, *MTERF10* and *MTERF11* levels were most responsive, and especially after 24 h of cold treatment *MTERF5, 10, 11*, and *12* transcript levels were reduced (Figure 5A). To confirm these data and to find other conditions under which the *MTERF*s of interest might be regulated, the Genevestigator Perturbations Tool (https://genevestigator.com/gv; Hruz et al., 2008) was employed on all deposited *A. thaliana* ATH1 arrays together with a 2-fold change filter and a *p*-value of < 0.05. An overview of all changes in *MTERF10, MTERF11*, and *MTERF12* mRNA levels in response to perturbations (relative to untreated controls) can be found in Supplementary Figures S1–S3. In Figure 5B selected conditions are shown which are associated with changes in temperature and light, and with salt and drought stress conditions. Levels of *MTERF10* mRNA were most susceptible to perturbation, being induced by light, raised after germination, and strongly reduced under drought conditions and various cold and high-light regimes, and on exposure to salt or ABA (Figure 5B).

### Knockdown of *MTERF10* or *MTERF11* alters sensitivity to salt stress

**Figure 6:**
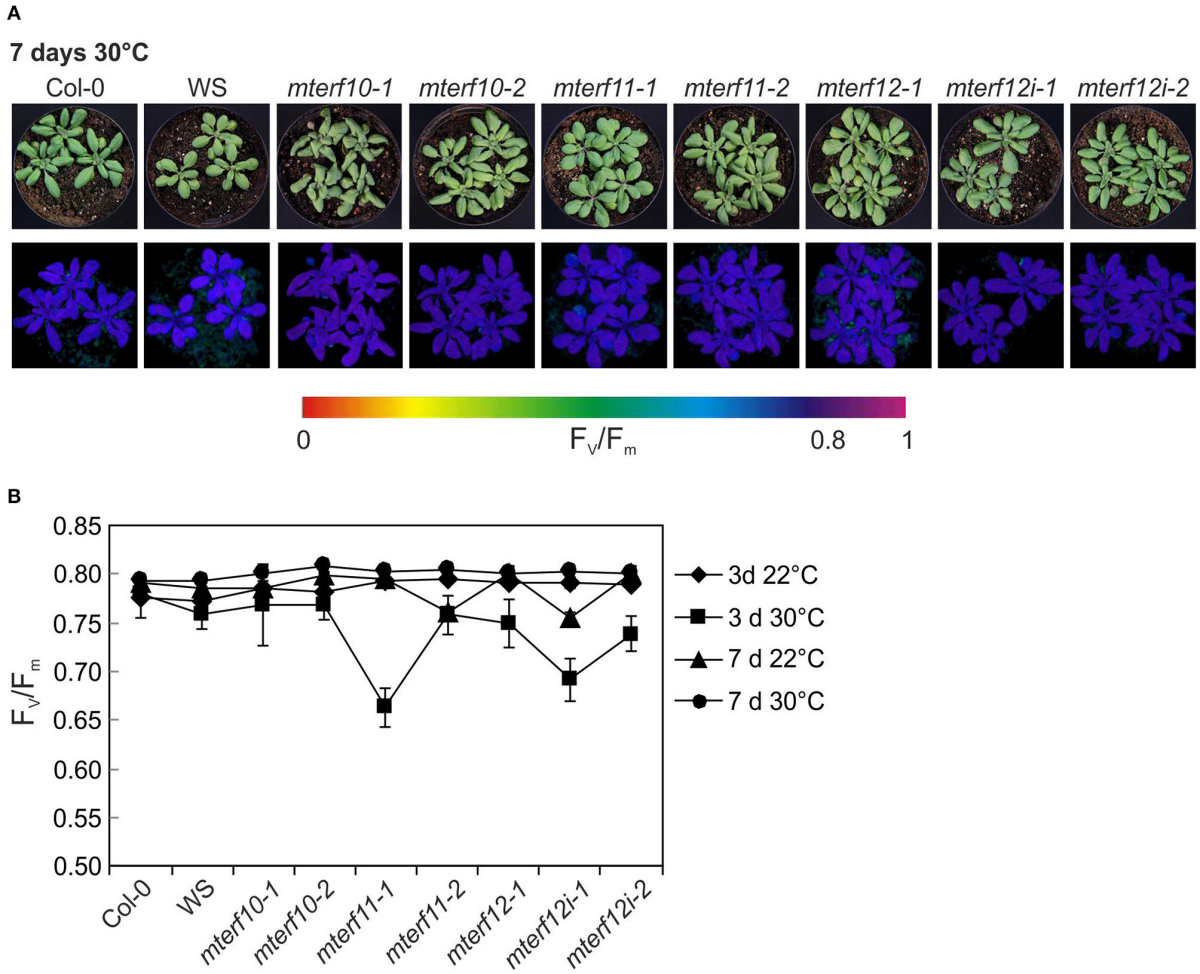
Behavior of wild-type (Col-0) and *mterf10*, -*11* and -*12* mutant plants under moderate heat stress of 30°C. **(A)** To score the phenotypes under moderate heat stress, plants were first grown for 3 weeks under normal growth conditions (100 μmol photons m^−2^s^−1^, 22°C) and then exposed to 30°C for 7 days. **(B)** The maximum quantum yield of PSII (F_v_/F_m_) of Col-0 and *mterf* mutant plants was determined after 3 and 7 days (d) in 30°C. The data are shown as mean values ± SD from 8 to 10 different leaves.

To experimentally probe the involvement of mTERFs in stress responses, 3-week-old WT and *mterf10*, -*11*, and -*12* mutant plants grown under standard conditions (22°C at 100 μmol photons m^−2^ s^−1^) were exposed for 7 days to moderate temperature stress (30°C, at a fluence rate of 100 μmol photons m^−2^ s^−1^) or moderate light stress (400 μmol photons m^−2^ s^−1^ at a temperature of 22°C). After 7 days of moderate temperature stress, the leaf petioles of WT and all *mterf* mutants were shortened, but otherwise all plants looked healthy (Figure 6A). After 3 days, F_v_/F_m_ was slightly reduced in all *mterf11* and *mterf12* mutant lines, but was restored to normal levels after 7 days (Figures 6A,B). Also after 7 days of moderate light stress, the leaf petioles of WT and all *mterf* mutants were shortened—albeit to a lesser extent. Furthermore, the edges of older leaves in all lines began to show signs of necrosis (Supplementary Figure S4A). After 1 h of moderate light stress, F_v_/F_m_ was slightly reduced in all lines (Supplementary Figure S4B). This reduction continued in the *mterf11-1* mutant after 2 and 4 h, but all lines recovered to the initial F_v_/F_m_ values after 96 h (Supplementary Figure S4B).

**Figure 7:**
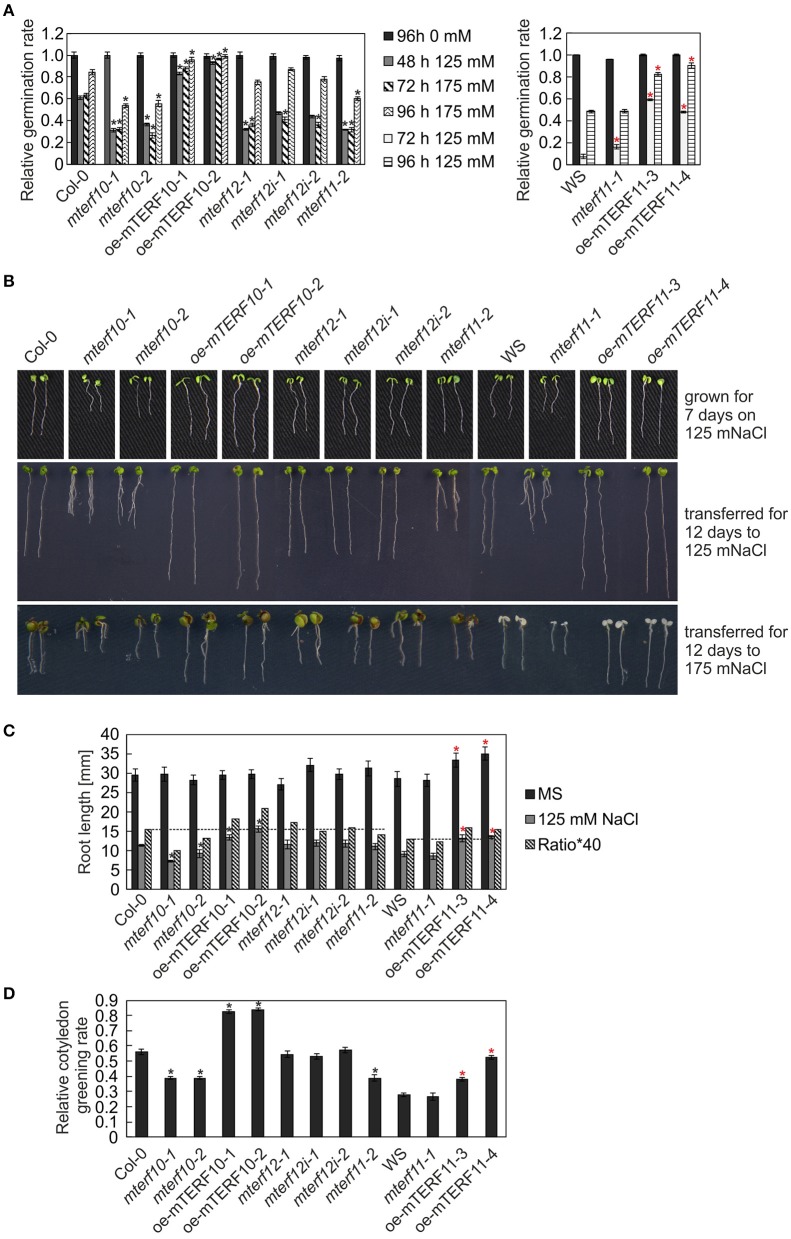
Responses of WT seedlings (WS for *mterf11-1* and oe-mTERF11 lines, and Col-0 for the remaining mutant lines), T-DNA (*mterf10-1* and -*2, mterf11-1* and -*2*, and *mterf12-1*), RNAi (*mterf12i-1* and -*2*) and overexpression lines (oe-mTERF10 and oe-mTERF11) to salt stress treatment under long-day conditions. **(A)** Seed germination was investigated on MS medium in the absence and presence of 125 and 175 mM NaCl. Radicle emergence was scored after indicated time points. Germination rates were calculated relative to the number of total seeds. **(B)** Phenotypes of 7-day-old WT and mutant seedlings were germinated on either MS medium supplemented with 125 mM NaCl (grown for 7 days on 125 mM NaCl), or on MS medium and transferred directly after radicle emergence for 12 days to MS medium supplemented with 125 mM NaCl (transferred for 12 days to 125 mM Nacl) or 175 mM NaCl (transferred for 12 days to 175 mM NaCl), respectively. The root lengths **(C)** and cotyledon greening rates of seedlings grown on 125 mM NaCl—displayed as the ratio of the number of green cotyledons to the total number of cotyledons **(D)** were determined after 7 and 5 days, respectively. The data in **(A,C,D)** represent mean values ± SD of three independent experiments, each performed with at least 100 seeds per treatment and genotype. Statistically significant differences (*t*-test; *p* < 0.05) between WT (Col-0 or WS) and corresponding mutant lines are indicated by an asterisk (black for Col-0; red for WS).

After 3 and 24 h of salt stress, *MTERF5* and *MTERF9* transcript levels were reduced to half of those in control conditions (Figure 5A), and indeed, *mda1* (*mterf5*) and *mterf9* seedlings are less sensitive to salt and osmotic stresses (Robles et al., 2012, 2015). Because *MTERF10* and *MTERF11* transcripts were reduced to an even larger extent than *MTERF5* and *MTERF9* RNAs following exposure to salt stress for 6 and 24 h (Figure 5A), we asked whether inactivation of *MTERF10* or *MTERF11* might enable the mutant plants to better tolerate salt stress. To this end, WT and *mterf* mutant lines were germinated on MS medium (control) and MS medium supplemented with 125 mM or 175 mM NaCl, and germination rates were scored after different time points (Figure 7A). All lines germinated to nearly 100% on the control MS medium. Germination rates of all lines with a Col-0 background grown for 48 h on MS medium supplemented with 125 mM NaCl or for 72 h on medium supplemented with 175 mM NaCl were very similar (Figure 7A). In the aforementioned conditions, germination rates of Col-0 seeds were approximately 60% (Figure 7A). The germination rates of *mterf10-1* and -*2, mterf11-2* and all *mterf12* seeds were all lower (ranging from 26 to 47%) than those of Col-0 seeds. However, after 96 h on MS medium supplemented with 175 mM NaCl the germination rates of *mterf12* seeds (76 to 87%) were comparable to that of Col-0 seeds (84%). Interestingly, *mterf10-1, mterf10-*2, and *mterf11-2* still displayed enhanced sensitivity to salt inhibition, with germination rates of 54, 55, and 61%, respectively (Figure 7A). WS seeds were very susceptible to salt stress and failed to germinate under the conditions used to investigate the Col-0 descendant lines. For this reason, a milder salt stress treatment was applied to all lines with a WS background. Still, after 72 h growth on MS medium supplemented with 125 mM, the germination rate of WS was only 7% and raised to 48% after 96 h (Figure 7A). Although the *mterf11-2* mutant was more sensitive to salt stress compared to Col-0, the germination rates of *mterf11-1* seeds were comparable to their corresponding WT (WS; Figure 7A).

**Figure 8:**
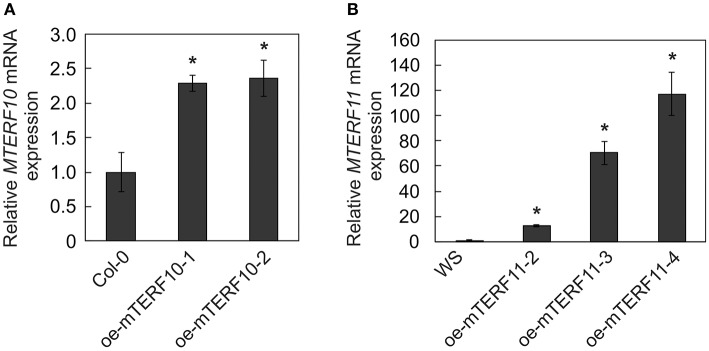
*MTERF10* **(A)** and *MTERF11* **(B)** transcript levels in Col-0 lines overexpressing *MTERF10* (oe-mTERF10) and WS lines overexpressing *MTERF11* (oe-mTERF11), respectively. Transcript levels were determined by real-time PCR analysis. Expression values are reported relative to the corresponding transcript levels in Col-0. The results were normalized with respect to the expression level of *At4g36800* (*RCE1*). Bars indicate standard deviations. Statistically significant differences (*t*-test; *p* < 0.05) between Col-0 and oe-mTERF10 lines and WS and oe-mTERF11 lines, respectively, are indicated by an asterisk.

To ascertain whether the altered activity of *MTERF10* was indeed responsible for the salt-stress phenotypes and whether overexpression of *MTERF11* might lead to enhanced salt-stress tolerance, *35S*:*MTERF10*:*MGFP5* and *35S:MTERF11:EGFP* constructs were introduced into Col-0 and WS, respectively, to generate oe-mTERF10 and oe-mTERF11 lines. Although *MTERF10* mRNA levels were only approximately 2.3-fold higher in oe-mTERF10-1 and oe-mTERF10-2 lines than in Col-0 (Figure 8A), these lines—with germination rates of approximately 85 and 95%, respectively, after 48 h on MS medium with 125 mM NaCl and 72 h on MS medium with 175 mM NaCl—were nevertheless resistant to the deleterious effect of salt (Figure 7A). This confirms that the salt sensitivity of *mterf10-1* and *mterf10-2* mutants is indeed caused by knockdown of the *MTERF10* gene. Moreover, we identified three oe-mTERF11 lines that displayed a high diversity of *MTERF11* transcript overaccumulation which ranged from 12- to 117-fold (Figure 8B). Two of these lines were challenged with salt stress, and actually displayed much higher germination rates than WS and therefore, enhanced salt stress tolerance (Figure 7A).

To investigate this further, the performance of *mterf* mutants and mTERF overexpression lines was investigated during post-germination development. As shown in Figures 7B,C, the root lengths of *mterf10-1* and -*2* seedlings challenged with 125 mM NaCl were significantly shorter compared to Col-0, while the root lengths of oe-mTERF10 seedlings were longer (Figure 7C). Moreover, compared to WS, overexpression of *MTERF11* results in longer root lengths, reflecting findings of the germination rates (Figure 7A). In addition, cotyledon greening—displayed as the ratio of the number of green cotyledons to the total number of cotyledons—of *mterf10-1* and -*2* seedlings was delayed by salt stress, while in contrast, overexpression of *MTERF10* or *MTERF11* enabled seedlings to display higher cotyledon greening rates than their corresponding wild types (Figure 7D).

**Figure 9:**
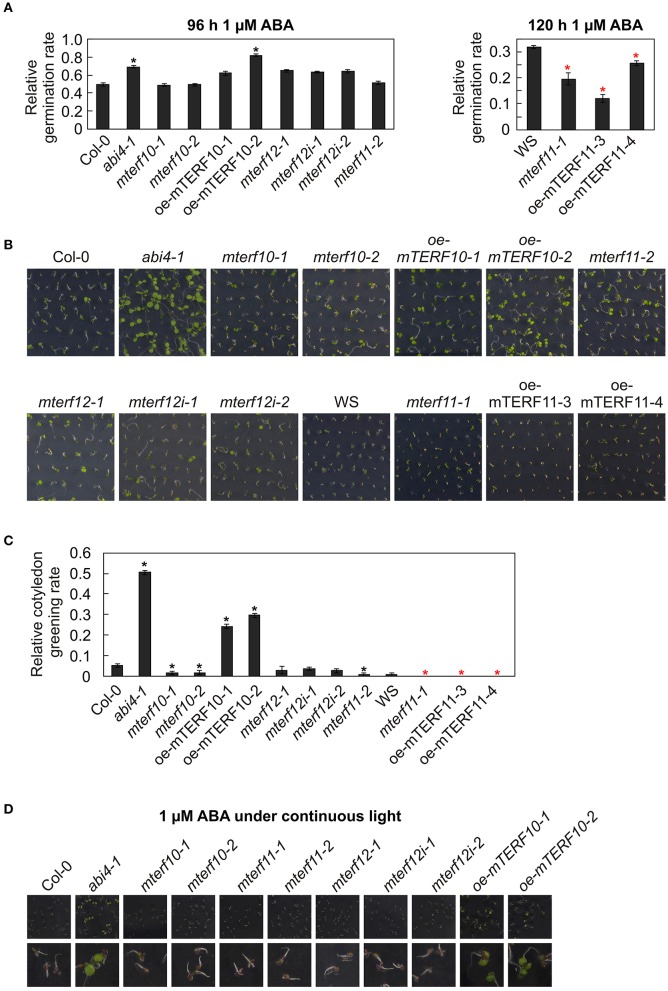
Responses of WT seedlings (WS for *mterf11-1* and oe-mTERF11 lines, and Col-0 for the remaining mutant lines), T-DNA (*mterf10-1* and -*2, mterf11-1* and -*2*, and *mterf12-1*), RNAi (*mterf12i-1* and -*2*) and overexpression lines (oe-mTERF10 and oe-mTERF11) and—as control—the *abi4-1* mutant to ABA treatment under long-day conditions **(A–C)** and under continuous light **(D)**. **(A)** Seed germination was investigated on MS medium in the absence and presence of 1 μM ABA. Radicle emergence was scored after indicated time points. **(B)** Phenotypes of 7-day-old seedlings grown on MS medium in the presence of 1 μM ABA. **(C)** The ratio of cotyledon greening was determined after 6 days. **(D)** Phenotypes of 7-day-old seedlings grown on MS medium in the presence of 1 μM ABA under continuous light. The pictures of the lower row are magnifications of the pictures above them. The data in **(A,C)** represent mean values ± SD of three independent experiments, each performed with at least 100 seeds per treatment and genotype. Statistically significant differences (*t*-test; *p* < 0.05) between WT (Col-0 or WS) and corresponding mutant lines are indicated by an asterisk (black for Col-0; red for WS).

ABA operates as a signal during developmental processes including seed germination, and moreover, in response to abiotic stresses including salt stress (Christmann et al., 2006). Furthermore, *A. thaliana* mutants in which the *ABI4* (*ABSCISIC ACID INSENSITIVE4*) gene has been inactivated are more salt tolerant than WT (Quesada et al., 2000; Shkolnik-Inbar et al., 2013). To investigate whether reduced *MTERF* transcript levels in the *mterf10*, -*11*, and -*12* mutant lines or overexpression of mTERF10 or mTERF11 alter ABA sensitivity, wild-type, *mterf*, oe-mTERF10, oe-mTERF11 and—as a control—*abi4-1* mutant seedlings were grown on MS supplemented with 1 μM ABA, and germination rates were scored after 96 h (lines with a Col-0 background) and 120 h (lines with a WS background). With a 69% germination rate, the control line *abi4-1* germinated better than Col-0 (49%; Figure 9A). All *mterf12* lines displayed a slightly, but not significantly, higher germination rate than Col-0, but *mterf10* and *mterf11* lines were as sensitive as Col-0 to ABA stress. Importantly, especially oe-mTERF10 lines were also less susceptible to ABA stress (Figure 9A), like they were to salt stress (Figure 7A). After 120 h on 1 μM ABA, WS germinated to 32%, and both *mterf11-1* and oe-mTERF11 lines displayed even lower germination rates (Figure 9A).

To follow this up, the phenotypes of seedlings grown on 1 μM ABA were examined after 7 days. Col-0 and WS seedlings displayed short roots and cotyledons had only started to emerge (Figure 9B). In contrast, *abi4-1* seedlings had longer roots and fully expanded cotyledons. All mutant lines with reduced *MTERF10*, -*11*, or -*12* transcript levels showed the same behavior as the wild types. However, the cotyledon phenotype of the oe-mTERF10 lines was comparable to that of the *abi4-1* mutant (Figure 9B). This was also manifested in the higher relative cotyledon greening rate of oe-mTERF10 lines (25 and 30%) compared to Col-0 (Figure 9C).

It appears that challenging Arabidopsis seedlings with ABA or salt stress under continuous light reduces germination efficiencies or cotyledon greening of Col-0 to a greater extent (Reyes and Chua, 2007; Chen et al., 2008; Hwang et al., 2015) compared to long-day conditions (Figure 7; Hu et al., 2013). To tackle the oe-mTERF10 ABA phenotype, we grew Col-0, *mterf* mutant lines and oe-mTERF10 lines in continuous light and a temperature of 20°C on MS medium (control) and MS medium supplemented with 1 μM ABA, and the phenotypes were scored after 7 days (Figure 9D). Because lines with a WS background were already very sensitive to ABA in long-day conditions, these lines were omitted. Indeed, Col-0 and *mterf* mutant seedlings displayed even shorter roots compared to long-day conditions and the cotyledons that had started to emerge did not turn green to this time point (Figure 9D). In contrast, *abi4-1* seedlings had longer roots and fully expanded green cotyledons. All mutant lines with reduced *MTERF10*, -*11*, or -*12* transcript levels showed the same behavior as Col-0. However, also in continuous light the phenotype of oe-mTERF10 lines was comparable to that of the *abi4-1* mutant, with longer roots and expanded green cotyledons (Figure 9D).

In sum, these results indicate that lower or higher *MTERF11* levels result both in increased ABA sensitivity. But, strikingly, higher *MTERF10* levels are associated with decreased sensitivity to ABA, which might in turn be linked to the higher salt tolerance of oe-mTERF10 lines.

## Discussion

*Arabidopsis thaliana* contains 35 mTERF proteins, of which seven have been investigated in more detail (reviewed in: (Kleine and Leister, 2015; Quesada, 2016)). Twenty-six of the 35 mTERFs have been sorted into groups based on their expression profiles and co-expression behavior (Kleine, 2012). The mTERF proteins that have been investigated in more detail are members of the “chloroplast” cluster (mTERF1, -4, -5, -6, and -9; the cluster itself contains 9 members) and the “mitochondrial” cluster (mTERF15 and -18; this cluster contains 7 members). In this study, we added to the inventory of characterized mTERFs and investigated all members of the “chloroplast-associated” cluster (mTERF10, -11, and -12).

In a fluorescence microscopy study of mTERF-GFP fusion proteins, 16 mTERFs were shown to be targeted to mitochondria, 11 to chloroplasts and one to the nucleus/cytosol (Babiychuk et al., 2011). That study revealed localization of mTERF4 to chloroplasts and mTERF6 to mitochondria. Later studies demonstrated that mTERF4 (Quesada et al., 2011) and mTERF6 (Romani et al., 2015) are in fact found in both mitochondria and chloroplasts. The mTERF proteins in the chloroplast-associated group were also assigned to the chloroplasts in the large-scale study cited above (Babiychuk et al., 2011). However, to confirm or extend these data, and also to define the sub-chloroplast localization of mTERF10, -11, and -12, we transiently transformed *A. thaliana* protoplasts with GFP fusion proteins. By co-transformation with a RAP-RFP fusion protein, which is a marker for the chloroplast nucleoid (Kleinknecht et al., 2014), we show that all members of the chloroplast-associated cluster are localized to chloroplast nucleoids (see Figure 1). Also the maize homologs of Arabidopsis mTERFs-2, -3, -4, -5, -7, -9, -16, and -27 were identified in enriched maize nucleoids (Majeran et al., 2012) and Arabidopsis mTERF8 was found in preparations of the plastid transcriptionally active chromosome (pTAC; Pfalz et al., 2006) which is related to the nucleoid (Pfalz and Pfannschmidt, 2013). The nucleoid houses proteins that are associated with DNA organization, replication and repair, and furthermore, are involved in transcription, and processing, splicing and editing of transcripts, suggesting that mTERFs participate in PGE (Majeran et al., 2012). In fact, the three plant mTERF proteins whose molecular functions are known do participate in PGE: mTERF4 is involved in chloroplast group II intron splicing (Babiychuk et al., 2011; Hammani and Barkan, 2014), mTERF6 promotes maturation of a chloroplast tRNA (Romani et al., 2015) and in *mterf15* mutants intron splicing of mitochondrial *nad2* transcripts is perturbed (Hsu et al., 2014). Because levels of 16 and 23S rRNAs, and thus chloroplast protein synthesis, are reduced in the *soldat10* mutant (Meskauskiene et al., 2009), it can be assumed that the mTERF1/SOLDAT10 protein is likewise involved in PGE.

Most of the previously characterized *mterf* mutants show phenotypes under normal growth conditions. Inactivation of mTERF1 (Meskauskiene et al., 2009) or mTERF4 (Babiychuk et al., 2011) is embryo lethal, the *mterf6* and *mterf15* knock-out mutants are seedling lethal (Romani et al., 2015) and retarded in growth and development (Hsu et al., 2014), respectively, and *mda1* (*mterf5*) and *mterf9* mutants are small and pale (Robles et al., 2012, 2015). We were unable to discern any phenotypic alterations in *MTERF12* RNAi lines, either under normal or challenging growth conditions. In fact, mTERF12 might not be a bona fide mTERF protein, because an analysis with the SMART tool fails to identify any mTERF domain in mTERF12 (see above). On the other hand, *MTERF12* is expressed at moderate to high levels in many developmental stages and organs (Kleine, 2012), and the mTERF12-eGFP fusion protein is localized to nucleoids (see Figure 1B). Therefore, while mTERF12 might not belong to the eponymous family, it may nevertheless be involved in PGE. Moreover, the residual amount of *MTERF12* (32% of WT transcript levels) present in *mterf12i-1* (see Figure 3A) may suffice to maintain a WT-like phenotype under all the conditions examined here, or alternatively we may not have hit upon the conditions required to provoke an abnormal phenotype in *mterf12i* lines. The latter possibility appears to be the more likely. For *MTERF12* mRNA levels are highest in pollen (Wang et al., 2008; Kleine, 2012) and the most pronounced change in *MTERF12* transcript level occurs in response to supplementation with nitrate (see Supplementary Figure S3), an intervention to which *mterf12i* lines were not subjected. Moreover, functional redundancy cannot be completely ruled out, although none of the three genes of interest originated from a duplication event (Kleine, 2012) and our phylogenetic tree (see Figure 4) and protein sequence comparisons (see above) do not strongly support this idea.

In addition to their pale color and growth-restricted phenotype, the *mda1* (*mterf5*) and *mterf9* mutants are less susceptible to salt and osmotic stresses, perhaps caused by reduced sensitivity to ABA (Robles et al., 2012, 2015). Notably, acclimation outputs are also altered by impairments in several other mTERF proteins (Meskauskiene et al., 2009; Quesada et al., 2011; Kim et al., 2012). Indeed, the emerging role of *A. thaliana* and maize mTERFs in acclimation and stress responses has already been noted and discussed (Zhao et al., 2014; Kleine and Leister, 2015; Quesada, 2016). This notion is especially of importance for crop plants, because plant development and growth is reduced in challenging growth conditions, leading finally to reduced yield. For this reason, several strategies have been tried to produce abiotic stress tolerance crop plants (Sah et al., 2016). With the aim to find a starting point to investigate stress tolerance in cotton, the response of cotton to abiotic stress treatments was studied with a cDNA library derived from samples treated with different stress conditions. Indeed, many transcripts for known stress-related genes, transcription factors and also mTERFs were enriched in this library (Zhou et al., 2016). Moreover, investigation of transcript level changes of six maize *MTERF* genes (maize *MTERF*2, -5, -11, -12, -13, and -28) in response to salt, ABA and NAA treatments showed an upregulation of *MTERF28* transcripts in all tested stress conditions, while *MTERF12* transcript levels were induced nearly 2-fold after salt stress treatment. This suggests that of these tested mTERFs, maize mTERF28 is the strongest candidate participating in all tested stress responses, while mTERF12 might be especially involved in salt stress responses (Zhao et al., 2014). Our results show that in contrast to the strong *mterf* mutant phenotypes which point to essential functions of several mTERFs (Meskauskiene et al., 2009; Babiychuk et al., 2011; Romani et al., 2015), lines with altered *MTERF10* or *MTERF11* levels show only conditional phenotypes, which become manifest under adverse growth conditions (see Figures 7, 9). Strikingly, under continuous light, lower *MTERF10* levels are associated with reductions in salt tolerance, while oe-mTERF10 lines are more tolerant of salt and ABA than wild-type plants. The altered responsiveness to ABA of *oge* and also plastid signaling mutants has been noted before. For example, the “mitochondrial PPR protein PENTATRICOPEPTIDE REPEAT PROTEIN FOR GERMINATION ON NaCl” (PGN; Laluk et al., 2011), the tetrapyrrole biosynthesis proteins GUN4 and GUN5 (Voigt et al., 2010) and the plastid-targeted PPR protein GUN1 (Cottage et al., 2010) alter responses to ABA. Notably, *gun1* mutants show only subtle growth phenotypes, but GUN1 is an important integrator of plastid signals (Koussevitzky et al., 2007). Like mTERF proteins, PPR proteins are typically targeted to chloroplasts or mitochondria, and alter expression of transcripts by influencing editing, turnover, processing or translation (Barkan and Small, 2014). With more than 400 members, the PPR protein family is one of the largest in land plants (Barkan and Small, 2014) and far exceeds the mTERF family in size. The enlargement of the plant PPR family has been linked to the evolution of a complex organellar gene expression system that is characteristic for plant organelles (Barkan and Small, 2014), and this is likely to be true of the mTERF protein family also (Kleine, 2012). Moreover, and in contrast to animals, plants are sessile organisms that are exposed to environmental changes and stresses. During evolution, the expansion and functional diversification of protein families has helped plants to successfully adapt to or tolerate different environmental stresses (Quesada, 2016). The mTERF family is a good case study for this phenomenon. With the characterization of an increasing number of plant mTERF proteins, it is becoming evident that they play a wide range of roles in mediating tolerance and acclimation to different abiotic stresses.

## Author contributors

Research was designed by TK. Research was performed by DX and TK. The manuscript was prepared by DX, DL, and TK.

### Conflict of interest statement

The authors declare that the research was conducted in the absence of any commercial or financial relationships that could be construed as a potential conflict of interest.

## Supplementary material

Supplementary Figure S1 Quantification of changes in *MTERF10* mRNA expression in response to perturbations as determined with the Genevestigator Perturbations Tool. The tool was employed on all deposited *A. thaliana* ATH1 arrays together with a 2-fold change filter and a *p*-value of < 0.05.

Supplementary Figure S2 Quantification of changes in *MTERF11* mRNA expression in response to perturbations as determined with the Genevestigator Perturbations Tool. The tool was employed on all deposited *A. thaliana* ATH1 arrays together with a 2-fold change filter and a *p*-value of < 0.05.

Supplementary Figure S3 Quantification of changes in *MTERF12* mRNA expression in response to perturbations as determined with the Genevestigator Perturbations Tool. The tool was employed on all deposited *A. thaliana* ATH1 arrays together with a 2-fold change filter and a *p*-value of < 0.05.

Supplementary Figure S4 Behavior of wild-type (Col-0) and *mterf10*, -*11* and -*12* mutant plants under moderate light stress of 400 μmol photons m^−2^ s^−1^. **(A)** To score the phenotypes under moderate heat stress, plants were first grown for 3 weeks under normal growth conditions (100 μmol photons m^−2^ s^−1^, 22°C) and then exposed to 400 μmol photons m^−2^ s^−1^ for 7 days. **(B)** The maximum quantum yield of PSII (F_v_/F_m_) of Col-0 and *mterf* mutant plants was determined after the indicated periods of exposure to a fluence of 400 μmol photons m^−2^ s^−1^. The data are shown as mean values ± SD from 8 to 10 different leaves.

Supplementary Table S1 Primers used in this study.
