# Supplementary figures and images for: Arabidopsis thaliana mTERF10 and mTERF11, but Not mTERF12, Are Involved in the Response to Salt Stress
Source: Front Plant Sci. 2017 Jul 14;8:1213. doi: 10.3389/fpls.2017.01213 (PMC5509804; doi:10.3389/fpls.2017.01213)

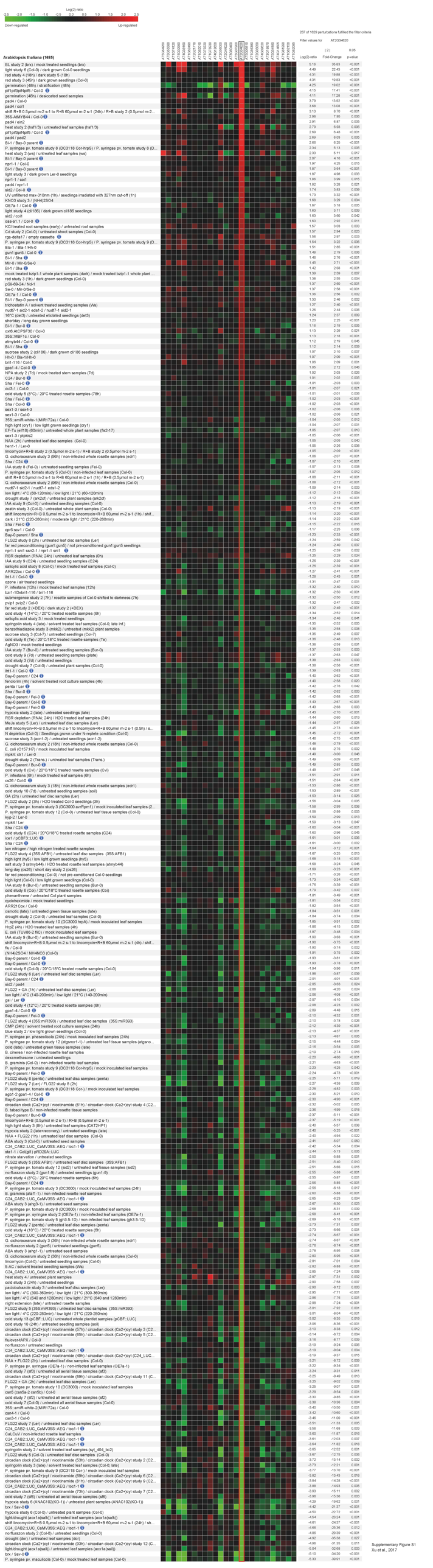

Supplement: Supplementary Figure S1 — Quantification of changes in MTERF10 mRNA expression in response to perturbations as determined with the Genevestigator Perturbations Tool. The tool was employed on all deposited A. thaliana ATH1 arrays together with a 2-fold change filter and a p-value of < 0.05. [file Image1.JPEG]

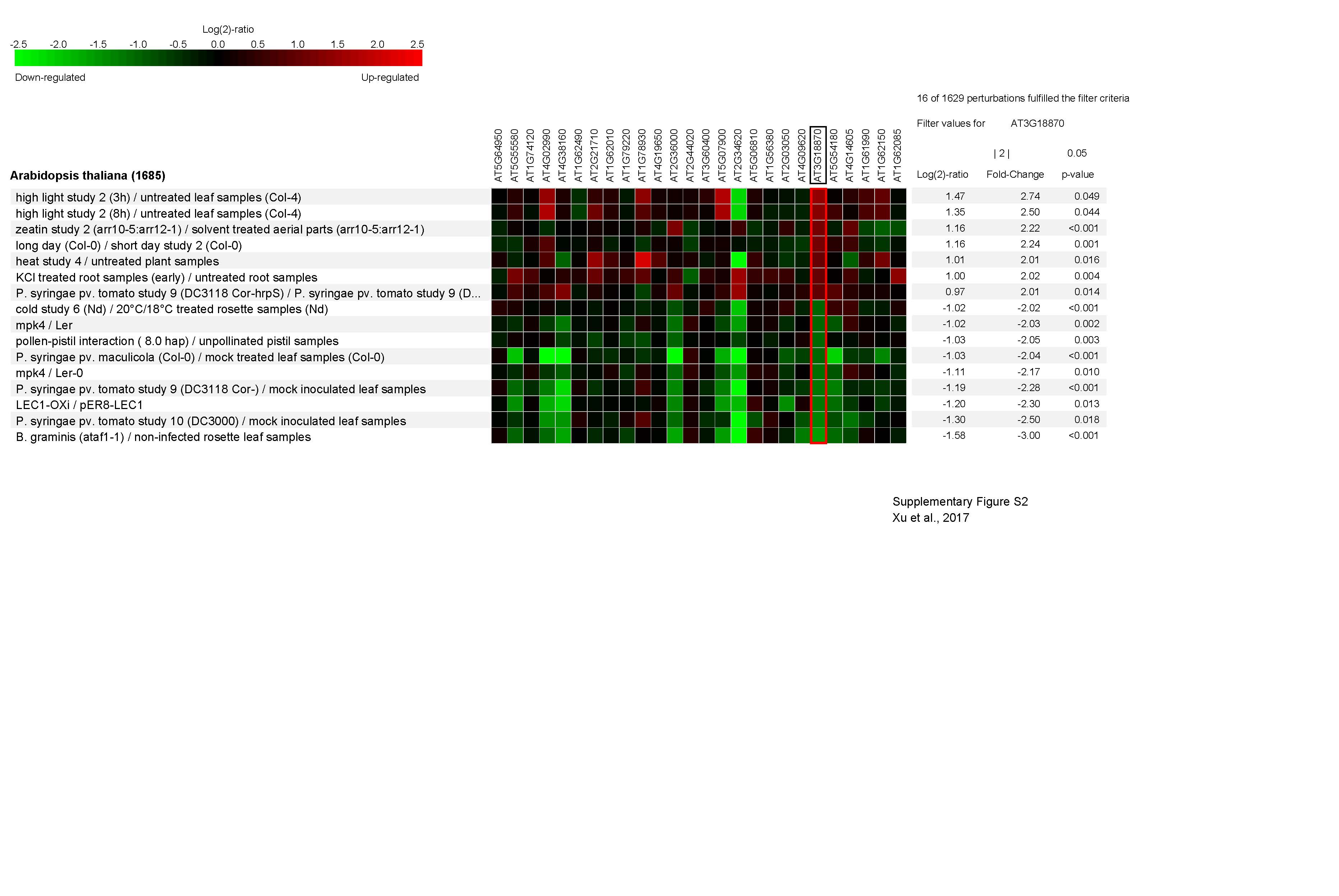

Supplement: Supplementary Figure S2 — Quantification of changes in MTERF11 mRNA expression in response to perturbations as determined with the Genevestigator Perturbations Tool. The tool was employed on all deposited A. thaliana ATH1 arrays together with a 2-fold change filter and a p-value of < 0.05. [file Image2.JPEG]

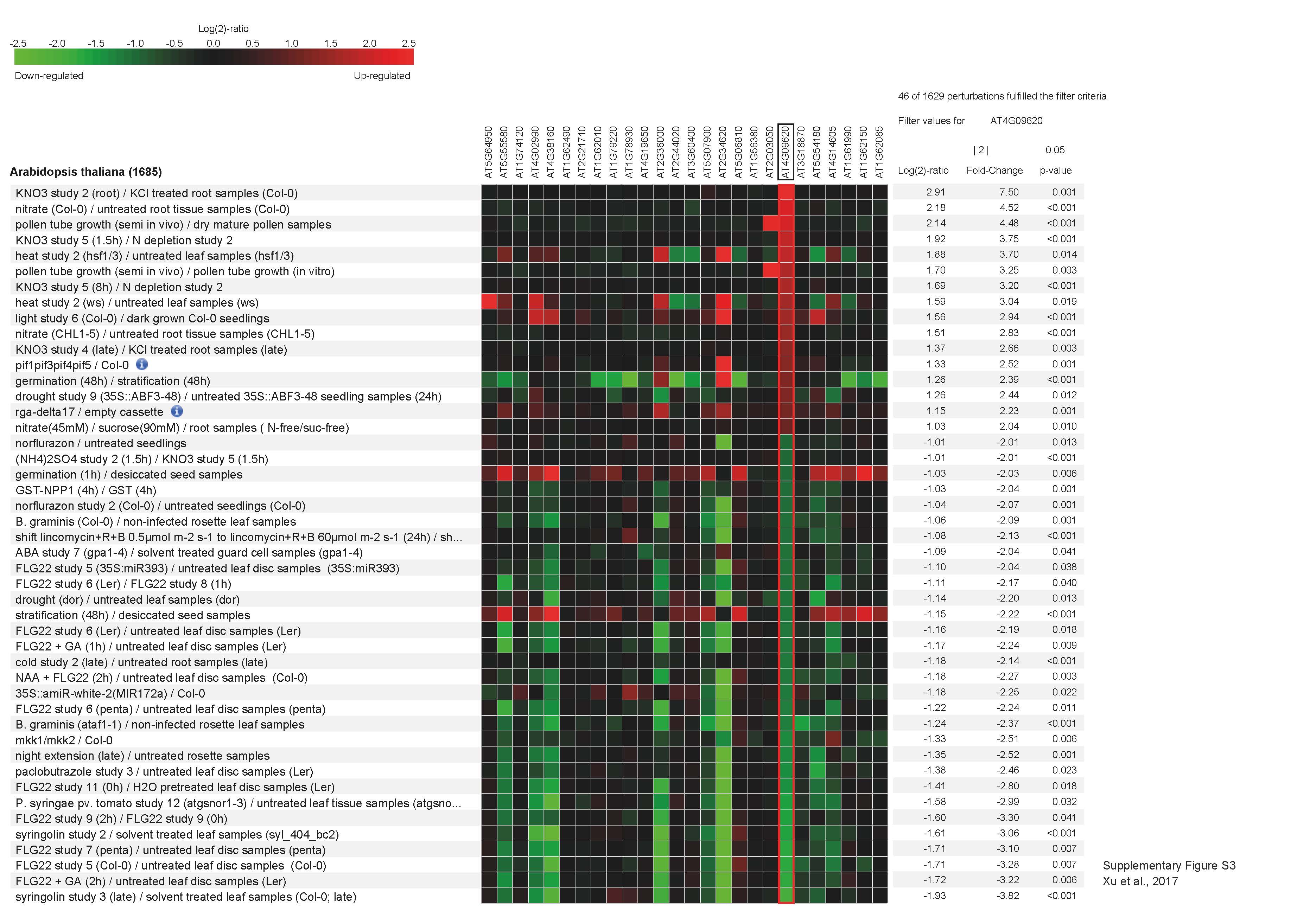

Supplement: Supplementary Figure S3 — Quantification of changes in MTERF12 mRNA expression in response to perturbations as determined with the Genevestigator Perturbations Tool. The tool was employed on all deposited A. thaliana ATH1 arrays together with a 2-fold change filter and a p-value of < 0.05. [file Image3.JPEG]

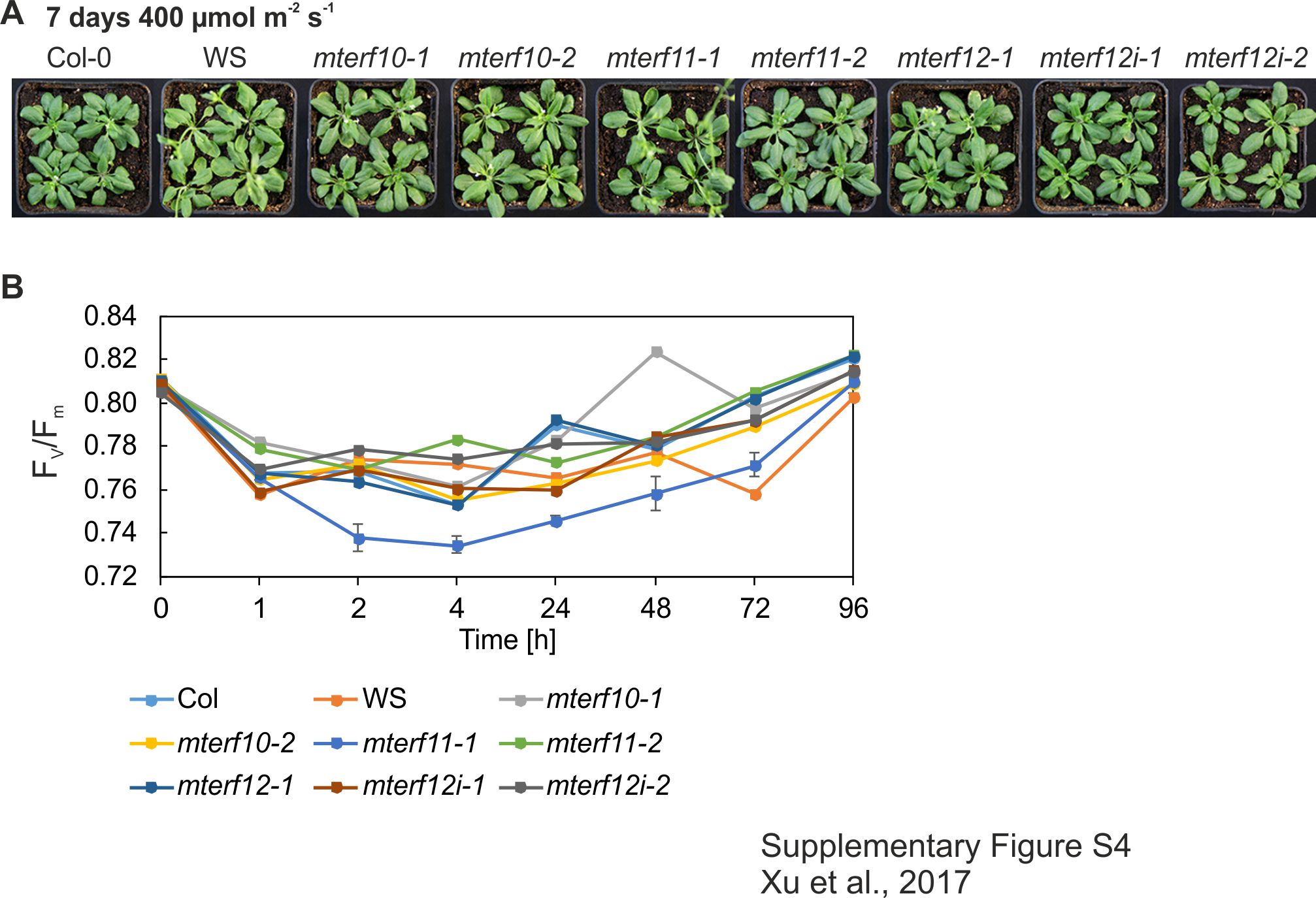

Supplement: Supplementary Figure S4 — Behavior of wild-type (Col-0) and mterf10, -11 and -12 mutant plants under moderate light stress of 400 μmol photons m−2 s−1. (A) To score the phenotypes under moderate heat stress, plants were first grown for 3 weeks under normal growth conditions (100 μmol photons m−2 s−1, 22°C) and then exposed to 400 μmol photons m−2 s−1 for 7 days. (B) The maximum quantum yield of PSII (Fv/Fm) of Col-0 and mterf mutant plants was determined after the indicated periods of exposure to a fluence of 400 μmol photons m−2 s−1. The data are shown as mean values ± SD from 8 to 10 different leaves. [file Image4.JPEG]
