# Supplementary material for: Arabidopsis thaliana mTERF10 and mTERF11, but Not mTERF12, Are Involved in the Response to Salt Stress
Source: Front Plant Sci. 2017 Jul 14;8:1213. doi: 10.3389/fpls.2017.01213 (PMC5509804; doi:10.3389/fpls.2017.01213)
Supplement: Supplementary Table S1 — Primers used in this study. [file Table1.DOCX]

**Supplementary Table S1**. Primers used in this study.

| **Name** | **Primer sequence (from 5’ to 3’)** |
| --- | --- |
| **Genotyping** |  |
| SALK_097699-LP | TTTACCCAACGCCTTTTTAGG |
| SALK_097699-RP | TCAGGAAAAGCCCTTTCTCTC |
| LbB1.3 (Genotyping of the SALK line) | ATTTTGCCGATTTCGGAAC |
| SAIL_12_A03-LP | TCGATGGAAACTGATTCAAAAAC |
| SAIL_12_A03-RP | ACATGGAGATCATTCGACAGG |
| LB3 (Genotyping of the SAIL line) | TAGCATCTGAATTTCATAACCAATC |
| FLAG_357F09-LP | ATCGTCACCGAGTTGAATCTC |
| FLAG_357F09-RP | GATCCCGTCTTCGATTTTCTC |
| FLAG-LB | CTACAAATTGCCTTTTCTTATCGAC |
| GABI_211D05-LP | AAATAAAATCGAACCGGGATG |
| GABI_211D05-RP | TCACTGGTTAGTTTCCGCAAC |
| GABI 407E04 LP | TCG ACT CAA ATC CGT CAA TTC |
| GABI 407E04 RP | GGA ACT TGA CAG GAA CAG AAG |
| GBKT (Genotyping of GABI KAT lines) | ATATTGACCATCATACTCATTGC |
| **Localization, overexpression and RNAi** | |
| AT4G09620-GST-attB1 | GGGGACAAGTTTGTACAAAAAAGCAGGCTATGGAGATGGTGGGAAAC |
| AT4G09620-GST-attB2 | GGGGACCACTTTGTACAAGAAAGCTGGGTGAAGATGTCCTTACGCCT |
| AT2G34620-NcoI-For | CCATGGCTGCAGCATTGAGCTCGTCT |
| AT2G34620-NcoI-Rev | CCATGGACGTTTGCCACTGAAGAGGG |
| mTERF11-18870-GFB-B1 | GGGGACAAGTTTGTACAAAAAAGCAGGCTATGGCTGTTATTGCTTCA |
| mTERF11-18870-GFB-B2 | GGGGACCACTTTGTACAAGAAAGCTGGGTGTGGCTTCCATTTTGAGTA |
| TERF12-4G09620GFP-B1 | GGGGACAAGTTTGTACAAAAAAGCAGGCTATGGAGATGGTGGGAAAC |
| TERF12-4G09620GFP-B2 | GGGGACCACTTTGTACAAGAAAGCTGGGTGAAACCGAACCCAACACCG |
| **Real-time** |  |
| AT4G36800-RCE1-RT-For | CTGTTCACGGAACCCAATTC |
| AT4G36800-RCE1-RT-Rev | GGAAAAAGGTCTGACCGACA |
| MTERF10-RT-new-For | GCCAAGACACTTAGAATCAATGGA |
| MTERF10-RT-new-Rev | ACACGTTTGCCACTGAAGAG |
| MTERF11-A-RT-new-For | TGAAGAGCTCAAGAAGTTTCC |
| MTERF11-A-RT-new-Rev | AACATTCTACTAAGCGAAACCC |
| MTERF11-B-RT-F | GAGGATAAGGCCAAGACATTGG |
| MTERF11-B-RT-R | AGATTATGGCTTATGGCTTCCA |
| mTERF12-4G09620-RT-F | AGCGTTTGGTCATATCCACTC |
| mTERF12-4G09620-RT-R | GCAACACAATCTCCTTTACAATCC |
